# Excessive Fluid in the Lumbar Facet Joint as a Predictor of Radiological Outcomes After Lateral Lumbar Interbody Fusion

**DOI:** 10.7759/cureus.30217

**Published:** 2022-10-12

**Authors:** Miguel Angel Roldan, Basar Atalay, Rodrigo Navarro-Ramirez, Sertac Kirnaz, Branden Medary, Fabian Sommer, Pravesh S Gadjradj, Roger Härtl

**Affiliations:** 1 Neurological Surgery, Weill Cornell Brain and Spine Center, New York, USA; 2 Neurological Surgery, Weill Cornell Brain and Spine Center, New York , USA; 3 Orthopedics/Spine, McGill, Montreal, CAN; 4 Neurological Surgery, Weill Cornell Medical College-New York Presbyterian Hospital, New York , USA; 5 Neurological Surgery, Weill Cornell Medical College-New York Presbyterian Hospital,, New York, USA

**Keywords:** facet joint, lateral lumbar interbody fusion, spine, minimally invasive spine surgery, decompression

## Abstract

Background

Preoperative segmental instability maybe a predictor of postoperative outcomes when treated with lateral lumbar interbody fusion (LLIF). An abnormal collection of fluid within the facet joint has been described as a sign of segmental instability. The potential relationship between this radiological sign and its prognostic relevance for indirect decompression (ID) has not been investigated.

Methods

Clinical and radiologic results from patients undergoing LLIF in a single institution between 2007 and 2014 were evaluated retrospectively. Patients were divided into two groups: those presenting with excessive fluid (EF) in the facet joints on T2-MRI and those with a normal amount of facet fluid with less than 1mm, which were controls. Radiological parameters were foraminal height, disc height, Cobb angle, and lumbar lordosis.

Results

A total of 21 patients (43 operated levels) were evaluated pre- and postoperatively. Mean disc height, mean foraminal height, and coronal Cobb angles were statistically significantly improved after LLIF. Only the EF group showed significant improvement in radiological markers after ID; the mean disc height improved from 5.5±2 to 8.8±1mm (p=0.001), mean foraminal height improved from 16.88±3 to 20.53±3mm (p=0.002), and the mean Cobb angle improved from 27.7±16 to 14±13 (p=0.018).

Conclusions

Patients undergoing LLIF with the radiological findings of EF in the facet joints demonstrated significant improvement in radiological outcomes of ID. Further studies should validate these findings in larger data sets.

## Introduction

Direct lateral lumbar interbody fusion (LLIF) is becoming increasingly more popular over the past decade [1,2]. Its effectiveness is linked to the concept of indirect decompression (ID) [3]. Identifying factors that can predict the success or failure of LLIF by ID aids in the decision-making process to choose the most effective and appropriate surgical treatment. In the past, some patient and procedure-related factors were identified that determine the success or failure rate of ID. Cage width was identified as the most significant procedure-related factor predicting ID [4]. Severe symptomatic lateral recess stenosis was identified as a patient-related radiological finding predicting the failure of ID [5]. Interestingly, a relationship could not be found between the severity of facet degeneration and the failure of ID [2]. However, an important radiological marker that has not been studied before is excessive fluid (EF) in the facet joints. EF in the facet joints is frequently associated with instability and hypermobility of segments. Its exact pathophysiology is not understood.

The relevant literature supports the idea that extensive facet fluid accumulation is an indicator of instability [6-7]. Therefore, a correlation between the presence of preoperative instability, excessive facet fluid accumulation, and surgical outcomes in ID could also exist. This study aims to explore the relationship between the surgical and radiological outcomes of ID in patients with and without hyperaccumulation of facet joint fluid.

## Materials and methods

Target population

**Figure 1:**
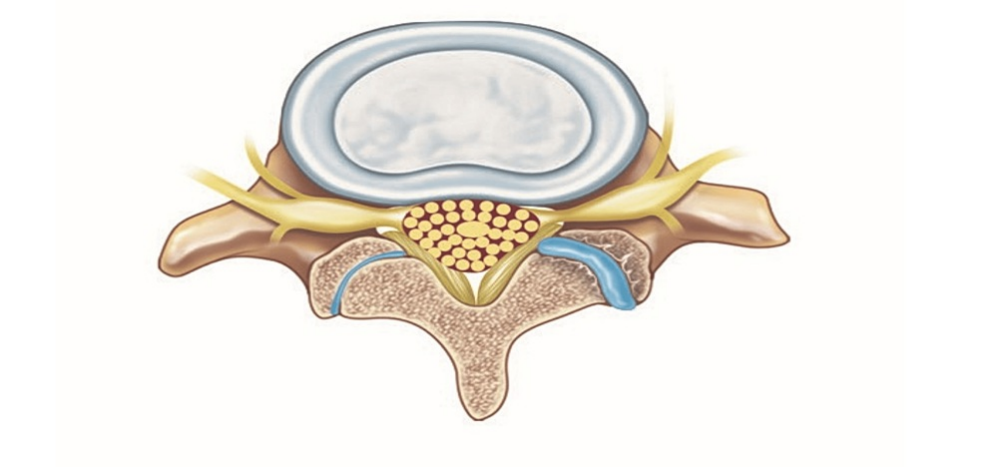
Radiological indicator of segmental instability is the presence of an abnormal amount of fluid collection in the facet joints of the suspected level. This illustration demonstrates the ruined joint with facet fluid accumulation on the right compared to the left normal facet joint. Copyright of the authors.

This study was designed as a retrospective case series. Patients undergoing LLIF surgery at the primary investigator’s institution for degenerative lumbar pathology between 2007 and 2014 with or without additional posterior transpedicular instrumentation were eligible. All patients had preoperative magnetic resonance imaging (MRI) and pre- and postoperative lumbar X-rays available. Patients undergoing direct decompression or those who had previously undergone surgery of the lumbar spine were excluded. Patients who had had an abnormal collection of fluid in the facet joints preoperatively were considered to have segmental instability (Figure 1). Patients without this EF were considered as stable controls. All other patients with other types of segmental instability such as pars defects, spondylolisthesis, or preoperative spine trauma were excluded from the study. This study and the data extraction were approved by the local institutional review board (IRB Protocol 19-12021199).

Radiological evaluation

**Figure 2:**
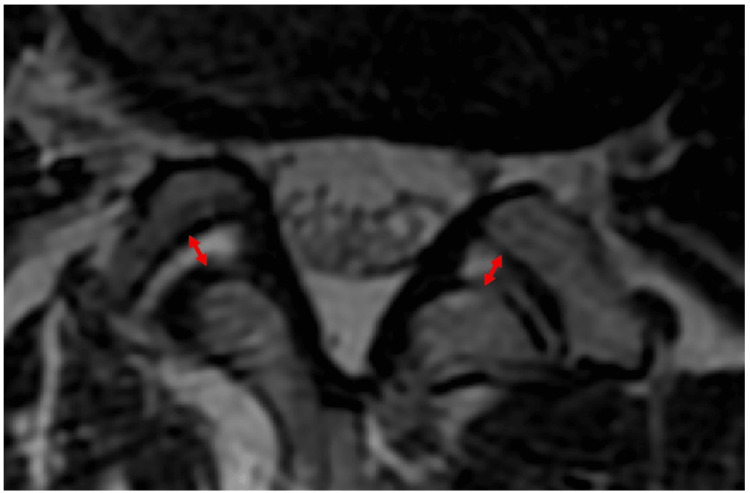
Presence of a cerebrospinal-like axial T2 MRI intensity liquid collection in the facet interarticular space equal to or larger than 1mm in its thickest cut is demonstrated as a radiologically significant atypical facet fluid accumulation. Measurements were performed bilaterally, and the average of both sides was calculated with Centricity Enterprise V3.0 software by drawing a perpendicular line from the mid facet joints' surfaces and measuring the distance between both bony landmarks.

Preoperative MRIs were collected retrospectively and reviewed for radiological findings of facet effusion. Schinnerer et al. defined the presence of a cerebrospinal-like axial T2-MRI intensity liquid collection in the facet interarticular space equal to or larger than 1mm in its thickest cut as a radiologically significant atypical facet fluid accumulation [7]. Thickness measurements were performed with Centricity Enterprise V3.0 software by drawing a perpendicular line from the mid facet joints' surfaces and measuring the distance between both bony landmarks (Figure 2). Measurements were performed bilaterally, and the average of both sides was calculated as is proposed in the literature [8].

**Figure 3:**
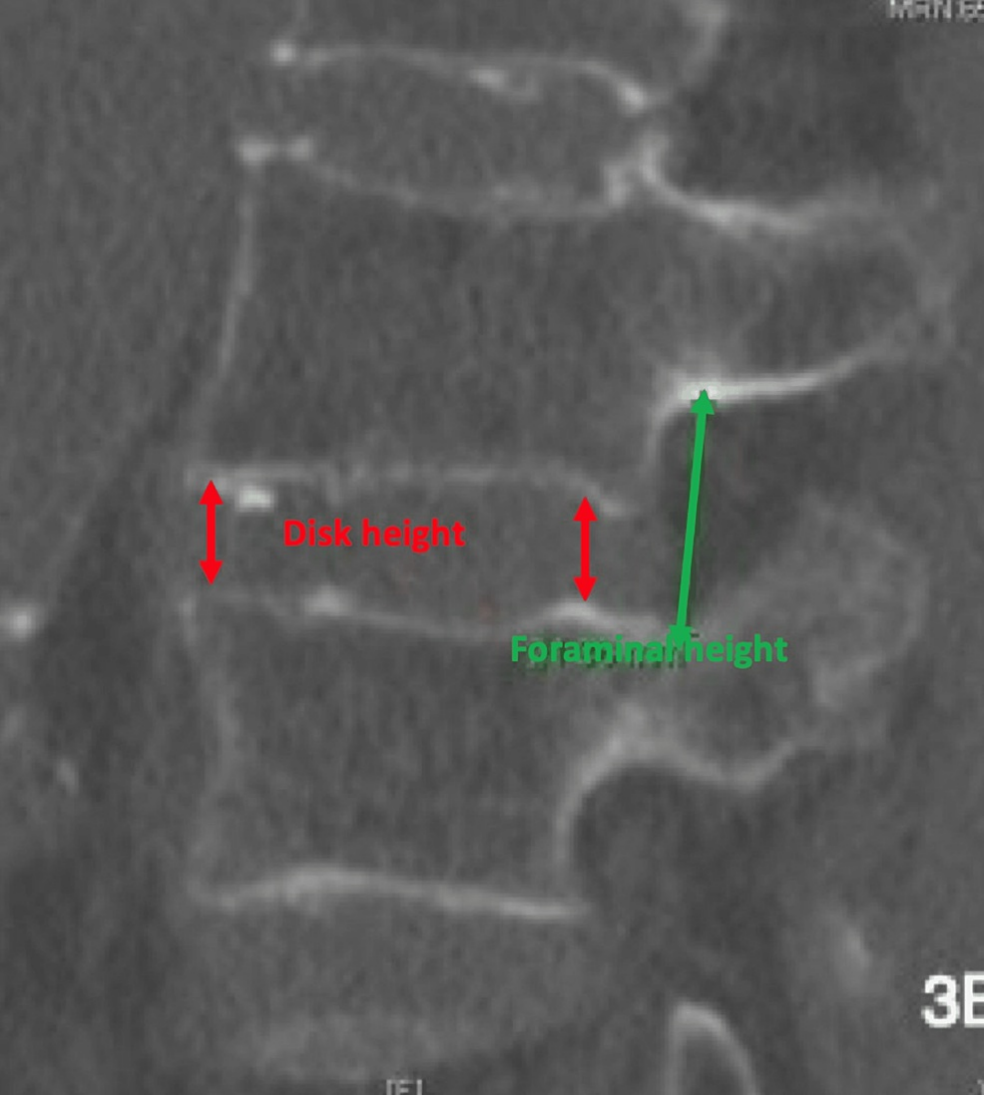
The disc height was measured as the distance between the caudal vertebra's upper surface and the lower surface of the cranial vertebra at the anterior (anterior disc height) and the posterior border (posterior disc height). The average between both was calculated (average disc height). The foraminal size was measured as the distance between the caudal pedicle's upper edge and the lower edge of the cranial pedicle. Measurement of the lumbar CT scan sagittal view is demonstrated.

Patients also underwent a lumbar intraoperative high-resolution fan beam computed tomography (CT) scan before and within 24 hours after surgery. Disc and foraminal heights were assessed from these scans. The disc height was measured as the distance between the caudal vertebra's upper surface and the lower surface of the cranial vertebra at the anterior (anterior disc height) and the posterior border (posterior disc height). The average between both was calculated (average disc height). The foraminal size was measured as the distance between the caudal pedicle's upper edge and the lower edge of the cranial pedicle (Figure 3). The average between both sides was calculated [9]. Cobb angles and Lumbar lordotic angles were measured on anteroposterior and lateral X-rays, respectively.

According to the literature, the cut-off point for “excessive” fluid was established at 1mm [10]. Any joint with a collection of more than 1mm of fluid was considered as EF that is leading to segmental instability. Consequently, the cut-off point of EF was determined as greater than or equal to 1mm.

Differences among radiological pre- and post-surgical variables for each group were evaluated separately. Subsequently, differences in the variations (pre- and post-surgical) between the controls and EF groups for each variable were further investigated.

Radiological measurements were completed by two neurosurgeons independently. Disc height, foraminal height, lumbar lordosis, and Cobb angles were compared between the controls and EF groups pre- and postoperatively.

Statistical analysis

Data were presented as mean ± standard deviation (Std). Since most of the data were not normally distributed (Kolmogorov-Smirnov test for Lilliefors significance correction and Shapiro-Wilk test), non-parametric tests were used. Differences in levels were compared. The differences among the groups were analyzed using the Wilcoxon signed-rank test. Computations were performed using IBM SPSS Statistics for Macintosh, Version 27.0 (IBM Corp. Armonk, NY). Graphs show means ± Std, with an asterisk (*) representing statistically significant differences (p<0.05) between groups.

## Results

A total of 21 patients for 43 operated levels met the inclusion and exclusion criteria. Of these, 11 were women and 10 were men, with an average age at the time of surgery of 64 ±11 years (range: 38-83 years). The baseline characteristics and radiological measurements are summarized in Table 1. At baseline, there were no differences between the EF group and controls.

**Table 1:** Demographics of all patients.

Variable	Frequency		
Gender	Male		10 (47.6%)
	Female		11 (52.4%)
Age at surgery (years)	64.7 ± 11(38.5-83.7)		
Height (cm)	167.0 ±12 (147-185)		
Operative blood loss (mm)	136.7±256 (50-1200)		
Operated level	L1-L2	2	5.3%
	L2-L3	8	21.1%
	L3-L4	14	36.8%
	L4-L5	13	34.2%
	T12-L1	1	2.6%
	Total	38	100.0%
Number of levels operated per patient	1	8	34.8%
	2	6	30.4%
	3	5	21.7%
	4	2	13.0%
	Total	21	100.0%

When assessing outcomes over the whole cohort, the disc heights showed a statistically significant increase from 5.2±2mm at baseline to 8±2mm postoperatively (p=0.001). Foraminal heights also showed a statistically significant increase from 16.1±3mm to 18.9±4mm (p=0.001), and coronal Cobb angles improved from 26.9±14 at baseline to 13.8±12 (p=0.005) postoperatively. Lumbar lordosis showed a nonsignificant increase from 41.2±16 to 43.5±15 (Table 2).

**Table 2:** Comparison of the preoperative and postoperative values of the radiological variables for the general sample.

Radiological variables measured overall (N=38 levels)			
Mean disc height (mm)	Preoperative	5.2±2	p=0.001
	Postoperative	8±2	
Mean foraminal height (mm)	Preoperative	16.1±3	p=0.001
	Postoperative	18.9±4	
Mean Cobb angle	Preoperative	26.9±14	p=0.005
	Postoperative	13.8±12	
Mean lordotic angle	Preoperative	41.2±16	p>0.005
	Postoperative	43.5±15	

The data were then divided into two groups as control (n=7) and EF (n=14), as previously described. The characteristics of each group are set out separately in Table 3.

**Table 3:** Comparison of the control group (n=7) and the excessive facet fluid accumulation group (n=14). Preoperative versus postoperative mean values for coupled samples were compared in the table.

Comparison of the control group (n=7) and the excessive facet fluid accumulation group (n=14)			
Control group radiological parameters			
Variable	Preoperative	Postoperative	Statistical significance
Mean disc height (mm)	4.3±3	6.0±2	p>0.05
Mean foraminal height (mm)	14.4±2	15.3±2	p>0.05
Mean Cobb angle	25.1±11	13.5±12	p>0.05
Mean lordotic angle	32.7±15	40.9±18	p>0.05
Excessive facet fluid accumulation group radiological parameters			
Variable	Preoperative	Postoperative	Statistical significance
Mean disc height (mm)	5.5±2	8.8±1	p=0.001
Mean foraminal height (mm)	16.8±3	20.5±3	p=0.002
Mean Cobb angle	27.7±16	14±13	p=0.018
Mean lordotic angle	45.1±15	44.7±14	p>0.05

**Figure 4:**
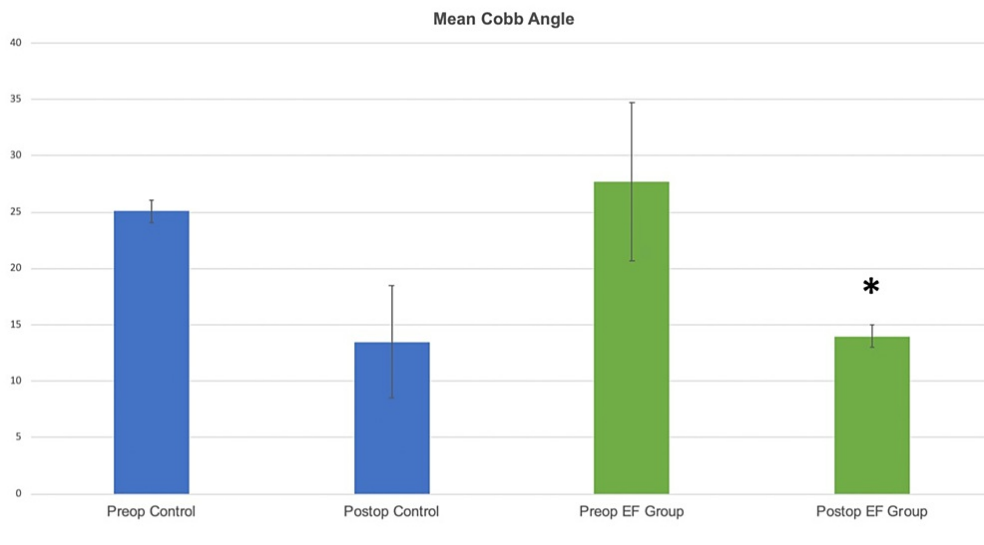
The preoperative mean Cobb angle in the control group improved from 25.1±11 to 13.5±12 (p>0.05) postoperatively and in the EF group it significantly improved from 27.7±16 preoperatively to 14±13 postoperatively (p=0.018).

The preoperative mean Cobb angle in the control group improved from 25.1±11 to 13.5±12 (p>0.05) postoperatively and in the EF group it significantly improved from 27.7±16 preoperatively to 14±13 postoperatively (p=0.018) (Figure 4).

**Figure 5:**
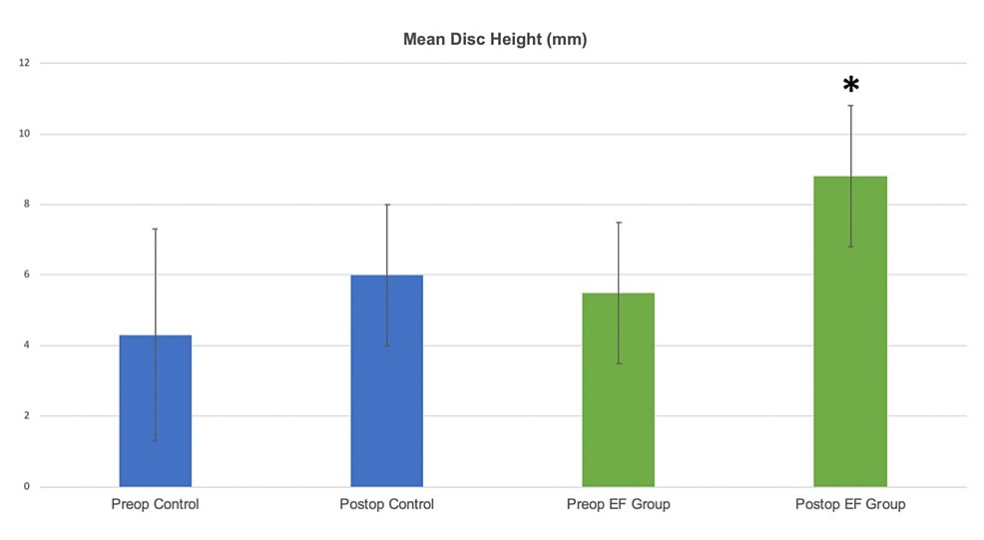
Radiological signs for ID in the control group for the mean disc height improved from 4.3±3mm to 6±2mm (p>0.05), and in the EF group it significantly improved from 5.5±2mm to 8.8±1mm (p=0.001).

The preoperative mean lumbar lordotic angles in the control group improved from 32.7±15 to 40.9±18 (p>0.05), and in the EF group, they changed minimally from 45.16±15 to 44.7±14 (p>0.05). Radiological signs for ID (foraminal and disc height) in the control group for the mean disc height improved from 4.3±3mm to 6±2mm (p>0.05), and in the EF group it significantly improved from 5.5±2mm to 8.8±1mm (p=0.001) (Figure 5).

**Figure 6:**
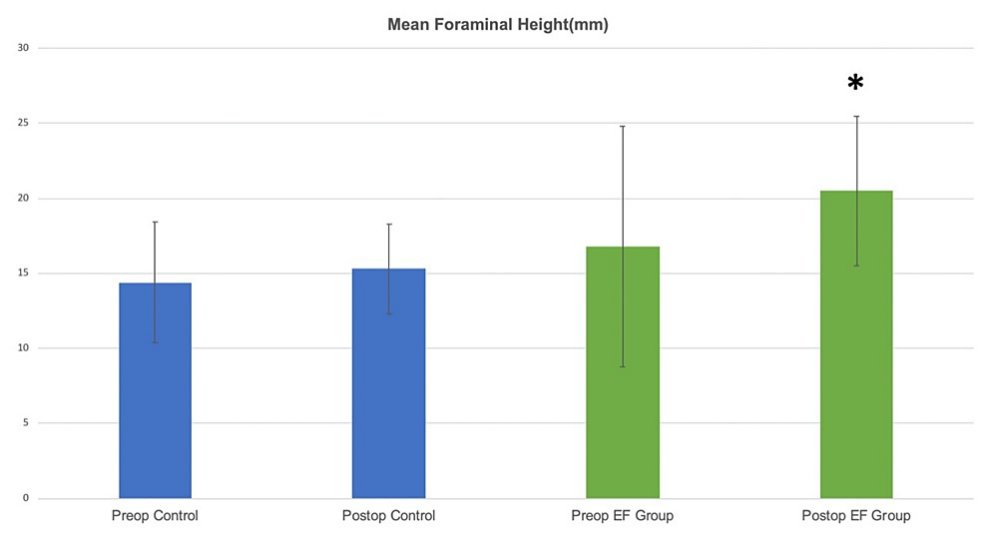
The mean foraminal height in the control group improved from 14.41±2mm to 15.38±2mm (p>0.05), and in the EF group it significantly improved from 16.88±3mm to 20.53±3mm (p=0.002).

Radiological outcome improvements in the EF group were significant compared to the improvements in the control group. The mean foraminal height in the control group improved from 14.41±2mm to 15.38±2mm (p>0.05), and in the EF group it significantly improved from 16.88±3mm to 20.53±3mm (p=0.002) (Figure 6).

## Discussion

The present research investigates whether preoperative abnormal facet joint fluid accumulation thicker than 1mm can be considered as a predictor of a good surgical outcome based on adequate radiological outcome improvements in disc heights, foraminal heights, and Cobb angles. EF collection indicates instability and the hypermobility of the segment [10-15]. This radiological finding suggests that the radiologically unstable hypermobile segment could receive greater benefit from LLIF. According to the current study results, a positive relationship exists between the presence of an atypical facet fluid collection, thicker than 1mm, and a significant increase in foraminal and disc height after LLIF. This association has not been previously investigated. In the current series, the control group exhibited improvement in radiological parameters, which did not reach a significance. However, in the EF group, significant improvement of the studied variables was demonstrated.

In previous publications, the authors of the current study differentiated between patient-related and procedure-related factors that determine the success of ID [5]. It was concluded that, among all these factors, the height of a cage was related to better ID and that severe lateral recess stenosis was related to a failure of ID [5-6]. The current findings now add to this literature and suggest that significant EF may be a predictor of instability.

LLIF technique has proven to be safe, reproducible, and effective in achieving excellent rates of fusion and symptomatic pain relief [10-16]. Different studies emphasize its effectiveness in alleviating neural compression, and the axial and radicular components of pain, even without carrying out a direct decompression [17,18]. For the radicular component, this effect is typically attributed to the concept of ID, which relies on augmentation of the surface area and volume of the anatomical compartments. Among the mechanical events that take part in ID, an increase in disc height, increase in the height and diameter of the foramen, "tension" of the posterior and anterior ligament (ligamentotaxis), and the stress and thinning of the ligaments inside the central canal have been cited [19].

Therefore, it seems reasonable to perceive that, given the described mechanism for ID, a spinal segment's hypermobility facilitates the surgical method. In addition, we may also assume that rigidity or fusion in a segment can lead to the failure of LLIF [20].

Currently, a strong consensus on radiological criteria for instability has not been established [14]. A previous study observed through a retrospective cohort that a relationship exists between finding a higher-than-expected/physiological fluid collection in the interarticular space of the facet joints and the presence of segmental instability [7]. Hyperaccumulation in the facet joint more accurately predicts the subsequent existence of hypermobility in dynamic X-rays than MRI. In fact, of the 16 segments included in the study that presented this sign, in only two the MRI detected spondylolisthesis. In contrast, dynamic X-rays showed instability in eight (50%) cases [7]. Conversely, in the control group consisting of 102 segments that did not have this hyperaccumulation, only one (0.9%) showed instability in the dynamic X-rays. Another study explored the relationship between degenerative spondylolisthesis and the existence of hyperaccumulation in the facet joints [9]. They found a strong correlation between the excessive facet fluid in T2 MRI and instability in dynamic lateral lumbar X-rays. The proposed biomechanical principle to explain the collection’s formation, and its relationship to instability is that segmental hypermobility would enable a separation of the facets in the supine position (when performing MRI), thus generating a real space that would be filled with synovial fluid, which would be reduced in standing position due to the effect of the axial load. Furthermore, this effect has been proved in studies that have performed MRI with axial load systems to check variations in the amount of liquid in the interarticular space between both positions with and without load [11].

The defining factors that can predict an adequate ID are of great interest for surgical planning, especially when deciding whether to perform an ID or fusion [12]. The literature review did reveal several factors that may lead to an LLIF "failure" [13], including the existence of a predominant bone component in lateral recess stenosis, severe central canal stenosis, and smaller diameter and sagittal area of the foramen. Various models of radiological analysis have been proposed to evaluate the effect of ID. Another study showed an increased risk for needing a secondary laminectomy, motivated by a failure of ID, in patients who had a higher preoperative VAS leg score, longer previous duration of symptoms, and/or lower gains in the disc and foraminal height [14]. In addition to the gain in the disc and foraminal height, the most recent studies pay attention to the sagittal section of the foramen and axial section of the central canal and whether the spinal canal is stenosed or not [15,16]. Volumetric models with three-dimensional measurements have also been proposed to study these changes [17,18].

In this study, a significant difference was not observed between the pre- and postoperative lordotic angles neither in the control or excessive facet fluid accumulation groups. EF in facet joints allowed distraction and ligamentotaxis, but these were not the only determinant for lumbar lordosis. Most of our cases were single level, and we used second-generation parallel cages. As a consequence, we did not achieve a significant change in lordotic angles. In addition, the main reason for this finding maybe the normal range of the preoperative lumbar lordotic angles (41.2±16) and the ID preserved the normal values (43.5±15). Furthermore, among all radiological parameters in this study, only lordotic angles were normal, and as the lordotic angles have not changed significantly, we may assume this value as a control parameter.

It was observed that patients who do present this EF radiological sign appear to experience a more noticeable radiological improvement, which can be considered as a predictor of success at the time of decision-making. As previously cited, many other factors must be analyzed to achieve good clinic results [19-25]. The results of the current study seem to corroborate other published studies with an exaggerated liquid collection and its relation to instability. Facet joint fluid accumulation of more than 1mm may be an indication for fusion, as the spine can be considered unstable.

The present work has some limitations, mainly depending on its retrospective and single-center design. Furthermore, a greater sample size would have been preferable, but the strict selection criteria obviously had an impact at that point. Nevertheless, a significant relationship between the main variables and the exposing factor could be found, which suggests that the power of the tests was valid. However, future research with prospective and randomized studies is necessary in order to confirm the existence of a possible relationship between facet fluid hyperaccumulation and facetogenic instability when predicting the success rate of ID.

## Conclusions

In our retrospective case series, patients undergoing LLIF who present with a fluid collection in the facet joints greater than 1mm at the operated level demonstrated significant improvement in radiological markers of ID. These results suggest that perhaps in patients with EF collection in the facet joints, a fusion procedure may lead to more radiological success. Therefore, a preoperative sign of EF collection in the facet joint may also aid surgeons in the preoperative surgical decision-making. Further prospective studies with a larger sample may be needed to confirm our findings.
